# Is there hope for HER2-positive colorectal cancer with Trastuzumab? A review of current evidence

**DOI:** 10.1097/MS9.0000000000004459

**Published:** 2025-11-25

**Authors:** Gbolahan Olatunji, Nicholas Aderinto, Emmanuel Kokori, Michael Awoyinfa, Oluwafemi Ajimotokan, Joan Oluwadamilola Ajayi, Nitin Narayan Rao, Ajayi Oluwatomisin Temidayo, Tejiri Napoleon, Chimezirim Ezeano, Sulaimon Olaide Bukky, Emmanuel Elorm Nortey-Adom, Sharon Aa-Inir Karbo, Israel Charles Abraham

**Affiliations:** a Department of Medicine, Johns Hopkins University Bloomberg School of Public Health, Baltimore, Maryland, USA; b Department of Medicine and Surgery, University of Ilorin, Ilorin, Nigeria; c Department of Medicine and Surgery, Ladoke Akintola University of Technology, Ogbomoso, Nigeria; d College of Medicine, University of Lagos, Lagos, Nigeria; e Department of Medicine, Babcock University Teaching Hospital, Ogun, Nigeria; f Department of Medicine, Kharkiv National Medical University, Kharkiv, Ukraine; g Department of Medicine, Johns Hopkins Medical Institution, Baltimore, Maryland, USA; h Faculty of Pharmacy, University of Lagos, Lagos, Nigeria; i Department of Medicine, Federal Medical Center, Abeokuta, Nigeria; j Department of Medicine, University of Nigeria Teaching Hospital, Ituku, Nigeria; k Accident and Emergency, Mid Cheshire NHS Trust, Cheshire, UK

**Keywords:** clinical trials, HER2-positive colorectal cancer, targeted therapy, Trastuzumab combinations

## Abstract

Trastuzumab combinations are emerging as a promising therapeutic strategy for HER2-positive colorectal cancer. This review investigates the current clinical data on efficacy and safety. Clinical trials have shown encouraging results, with objective response rates (ORRs) ranging from 22.2 to 46% and complete remission achievable in some patients. Additionally, these combinations demonstrate potential for improved overall survival (OS). However, optimizing therapeutic strategies remains crucial. Future research should tailor treatment regimens based on tumor biology and individual patient characteristics. Investigating ctDNA genotyping as a reliable method for identifying responders and exploring the role of Rat Sarcoma Virus (RAS) mutations and other molecular markers are key areas for optimizing patient selection and treatment efficacy. Understanding resistance mechanisms is vital for developing combination therapies that overcome these pathways.

## Introduction

Colorectal cancer (CRC) remains a significant global health burden, with an estimated 1.93 million new cases and 940 000 deaths annually according to the 2022 GLOBOCAN report^[1]^. While advancements in screening and surgical techniques have improved early detection and treatment outcomes, CRC poses a significant challenge, particularly for patients with advanced stages^[2]^. Conventional therapies for advanced CRC, including surgery, chemotherapy, and radiation, can be limited by drug resistance and severe side effects, which can significantly impact patient quality of life^[3]^.

**HIGHLIGHTS**

- Clinical trials have shown encouraging results, with ORRs ranging from 22.2 to 46% and complete remission achievable in some patients.
- Additionally, these combinations demonstrate potential for improved OS.
- However, optimizing therapeutic strategies remains crucial.
- Future research should focus on tailoring treatment regimens based on tumor biology and individual patient characteristics. Investigating ctDNA genotyping as a reliable method for identifying responders and exploring the role of RAS mutations and other molecular markers are key areas for optimizing patient selection and treatment efficacy.
- A deeper understanding of resistance mechanisms is also vital for developing combination therapies that overcome these pathways.

Targeted therapies offer a promising avenue for improved treatment outcomes in CRC. These therapies exploit specific molecular alterations within cancer cells, allowing for a more precise and potentially less toxic approach than traditional chemotherapy. One such targeted therapy gaining interest is Trastuzumab, a monoclonal antibody specifically targeting the human epidermal growth factor receptor 2 (HER2) protein^[4]^. Its application in HER2-positive CRC (3–5% of metastatic cases) offers a targeted, potentially less toxic alternative to conventional therapies^[5,6]^. Trastuzumab has shown remarkable success in HER2-positive breast cancer by blocking signaling pathways and inhibiting tumor growth^[6]^.

HER2 amplification in a subset of CRC patients presents a potential Achilles’ heel that Trastuzumab could exploit with its targeted action on this protein. Moreover, the established success of Trastuzumab in combating HER2-positive breast cancer offers a compelling argument for deploying it against a similar foe in CRC. For patients with advanced-stage HER2-positive CRC, existing treatment options are often limited and come with significant side effects. Trastuzumab offers the potential for a more targeted and potentially less toxic approach, making it a promising candidate for further investigation. This review aims to evaluate the current evidence regarding Trastuzumab’s efficacy and safety in treating HER2-positive CRC. This paper adheres to the TITAN guidelines^[7]^.

## Methods

**Figure 1.**
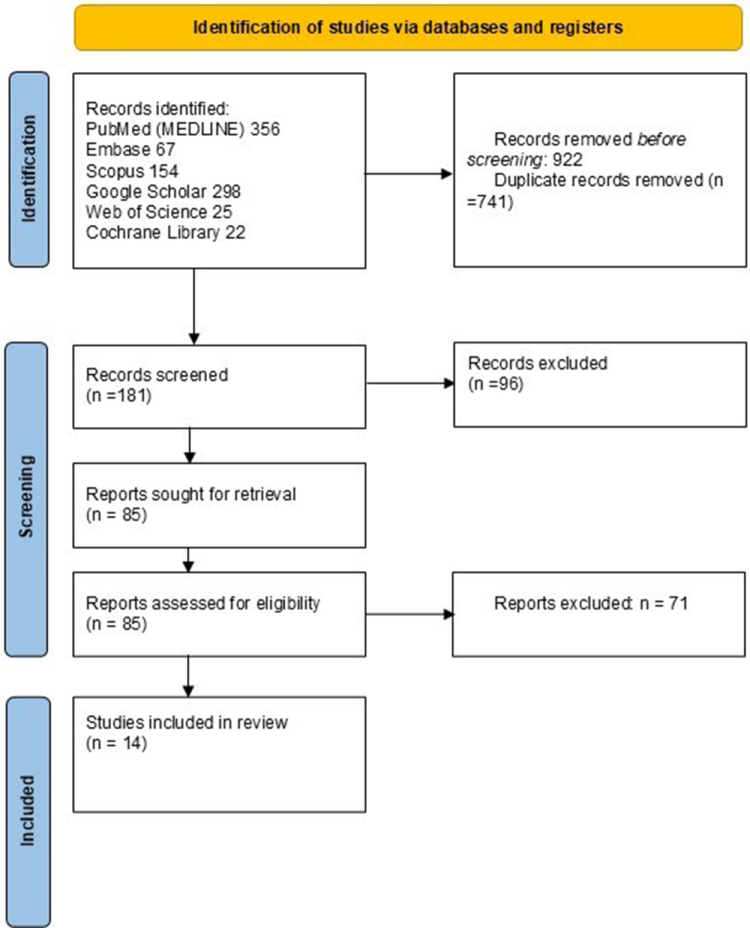
Screening process.

This review aims to evaluate the current evidence on the efficacy and safety of Trastuzumab as a targeted therapy for HER2-positive CRC – Table 1. A search strategy was employed to identify relevant studies published in English. We searched electronic databases, including PubMed, MEDLINE, Google Scholar, Web of Science, Embase, SCOPUS, and the Cochrane Library from the databases’ inception to April 2025. The search terms included a combination of Medical Subject Headings terms and keywords such as “Trastuzumab,” “HER2,” “colon cancer,” “colorectal cancer,” “targeted therapy,” “efficacy,” and “safety.” Boolean operators (AND, OR, NOT) were used to refine the search and ensure retrieval of the most relevant articles – Figure 1.

**Table 1:** Trastuzumab used as a single therapy in HER2-positive colorectal cancer

Author (year)	Study design and population	Intervention	Efficacy outcomes	Adverse events/safety	Other notable findings
Yoshino *et al* (2023)	Phase II, open-label, multicenter trial; 86 patients (Group A: 53, Group B: 15, Group C: 18)	Trastuzumab deruxtecan (T-DXd) 6.4 mg/kg every 3 weeks until death, withdrawal, unacceptable toxicity, or progression	Group A: Objective response rate (ORR) 45.3%; no response in Groups B or C. Median duration of response (DoR): 7 months; progression-free survival (PFS): 6.9 months; overall survival (OS): 15.5 months	Drug-related interstitial lung disease/pneumonitis (8 cases); most common grade ≥3 events: anemia and neutropenia	HER2 positivity did not influence serum exposure of T-DXd, DXd, or total anti-HER2 antibody after one cycle
Singh Raghav *et al* (2023)	Phase II, multicenter, randomized trial; 122 patients	T-DXd administered at 5.4 mg/kg (*n* = 82) or 6.4 mg/kg (*n* = 40) every 3 weeks until progression or withdrawal	Confirmed ORR: 37.8% (5.4 mg/kg) and 27.5% (6.4 mg/kg); median PFS: 5.8 and 5.5 months; median DoR: 5.5 months for both groups	52.4% experienced grade ≥3 treatment-emergent adverse events (TEAEs); one treatment-related death; drug-related interstitial lung disease in 12 patients (7 in 5.4 mg/kg group, 5 in 6.4 mg/kg group)	Antitumor activity observed regardless of RAS mutation status in patients pretreated with anti-HER2 therapy; 5.4 mg/kg dose favored for safety

Study selection: After the initial search, retrieved articles were screened based on title and abstract using predetermined inclusion and exclusion criteria. Studies were included if they:

- Investigated the use of Trastuzumab in the treatment of HER2-positive CRC.
- Reported on clinical outcomes, such as efficacy [objective response rate (ORR), progression-free survival, overall survival (OS)] or safety (adverse events).
- Were primary research studies (clinical trials, cohort studies).

Studies were excluded if they:

- Focused on a different cancer type or population.
- Employed irrelevant methodologies, such as case reports or animal studies.
- Did not provide sufficient data on efficacy, safety, or mechanisms of action.

Two reviewers independently extracted relevant data from the included studies using a standardized data extraction form. Extracted information included study design, patient characteristics, treatment regimens, efficacy outcomes, safety profiles, and any reported limitations. Disagreements between reviewers were resolved through discussion or by consulting a third reviewer. The extracted data were organized and categorized based on emerging themes. Risk-of-bias was not formally assessed due to the review’s focus on summarizing clinical outcomes rather than meta-analysis.

## Results

There were 14^[8–21]^ included studies that matched our inclusion and exclusion criteria – Table 2. A total of 754 patients were included in these trials. In total, 12 of the 14 (85.7%) included studies were phase 2 trials (10) (83.3%) of the phase 2 studies were open-label clinical trials, while 2 (16.7%) of the studies were randomized clinical trials. The other two studies (14.3%) were retrospective (one was a retrospective cohort study, while the other was a retrospective observational study).

**Table 2:** Trastuzumab in combination therapy for HER2-positive colorectal cancer

Author (year)	Study design and population	Intervention	Key efficacy outcomes	Adverse events/safety	Notable findings
Yang *et al* (2022)	Retrospective cohort; 63 patients. Group A: with trastuzumab; Group B: without	Combination chemotherapy ± targeted drugs	OS: 48.9 months (Group A) vs. 32.2 months (Group B) (*P* < 0.05). Application timing (first–fourth line) NS	Most frequent: GI events, myelosuppression (mainly from chemo)	Unresectable primary lesion, right-sided CRC, male sex = poor prognostic factors. Resection and sex = independent prognostic factors
Spiekman *et al* (2024)	Phase II multicenter; 27 patients	Trastuzumab (8 → 6 mg/kg q3w) + Pertuzumab (840 → 420 mg q3w)	ORR 46%; median DoR 8.4 months; PFS 4.3 months; OS 8.2 months	27 grade ≥3 events (16 pts); diarrhea most common; 1 death (unrelated)	Whole-genome sequencing (WGS) explained T-DXd resistance in ≈ 30%
Narita *et al* (2022)	Retrospective observational; 75 pts (MyPathway vs. real-world controls)	Trastuzumab + Pertuzumab (as in MyPathway trial)	HR for OS = 0.729 (95% CI 0.184–3.900) favoring MyPathway group	As reported in MyPathway trial	Sensitivity analysis confirmed robustness of OS benefit
Nakamura *et al* (2021)	Phase II single-arm multicenter; 44 pts (tissue+, ctDNA+)	Trastuzumab + Pertuzumab (q3w). Control n = 14 (SCRUM-Japan)	ORR 30% (tissue+); 28% (ctDNA+); 1 CR each. DoR 8.1–12.1 months; PFS 3.1–4.0 months; OS 8.8–10.1 months	80% experienced AEs; 3 grade 3 drug-related events; no deaths	ctDNA genotyping ≈ tissue accuracy; early ctDNA decline → better response
Meric-Bernstam *et al* (2019)	Phase IIa basket trial; 57 pts	Trastuzumab + Pertuzumab (q3w).	ORR 32% (1 CR, 17 PR); median DoR 5.9 months; PFS 2.9 months; OS 11.5 months	37% had grade 3–4 TEAEs (hypokalemia, abdominal pain). No deaths	KRAS wild-type mCRC → better responses and survival
Strickler *et al* (2023)	International Phase II RCT; 117 pts (Group A *n* = 45, B *n* = 41, C *n* = 31)	Tucatinib 300 mg daily + Trastuzumab (q3w) vs. Tucatinib alone	ORR 38.1% (A + B); 3 CRs, 29 PRs	Diarrhea most common; HTN most frequent grade 3+ event; no treatment-related deaths	Disease progression = main cause of death
Siena *et al* (2015)	Phase II trial; 23 pts	Trastuzumab + Lapatinib (1000 mg daily)	ORR 35%; responses lasting 8–55+ weeks; median TTP 5.5 months	Mostly grade ≤2 AEs (diarrhea, fatigue, rash)	Decrease in HER2+ ctDNA and plasma HER2 ectodomain correlated with response
Siena *et al* (2016)	Phase II (HERACLES trial); 33 pts	Trastuzumab (4 → 2 mg/kg weekly) + Lapatinib (1000 mg daily)	ORR 31% (9 PR, 1 CR); disease control 61%	6 grade 3 AEs (elevated bilirubin, rash, fatigue); no drug-related SAEs	Results consistent with HERACLES trial findings
Sartore-Bianchi *et al* (2020)	Phase II single-arm; 31 pts	Pertuzumab + Trastuzumab Emtansine (T-DM1) (q3w)	ORR 9.7%; stable disease 67.7%; PFS 4.1 months	Nausea, fatigue (mostly grade ≤2); thrombocytopenia (grade 3 in 2 pts)	Higher IHC scores → better ORR/stable disease ≥ 4 months
Tosi *et al* (2020)	Phase II trial; 35 pts; 82-month follow-up	Trastuzumab (4 → 2 mg/kg weekly) + Lapatinib (1000 mg daily)	ORR 28% (8 PR, 1 CR); PFS 4.7 months; OS 10 months; 1 pt maintained CR > 7 years	Mild AEs (diarrhea, rash, fatigue); 10 grade 3; no grade 4–5	19% developed CNS recurrence at 6.7 years
Xu *et al* (2023)	Phase II open-label multicenter; 21 pts	Trastuzumab (q3w) + Irinotecan (120 mg/m^2^ days 1 and 8).	ORR 33.3%; stable disease 52.4%; PFS 4.3 months	Mostly grade 1–2; grade 3 (diarrhea, UTI, neutropenia, leukopenia). No deaths	Resistance drivers: cell-cycle, PI3K/AKT, HER2, MAPK pathways
Fu *et al* (2023)	Phase II open-label single-arm multicenter; 20 pts	Trastuzumab (q3w) + Pyrotinib (400 mg daily)	ORR 22.2%; disease control 61.1%; PFS 3.4 mo. RAS WT subgroup: ORR 33.3%; DCR 83.3%	Mostly mild diarrhea; no grade 4–5 AEs	Supports Pyrotinib + Trastuzumab as RAS WT-specific option

The included studies for Trastuzumab used as a single therapy demonstrated varying ORRs and progression-free survival outcomes, ranging from 27.5 to 45.3% and 5.5 to 6.9 months, respectively. As investigated by the selected studies, combination therapies showed promising ORRs and median progression-free survival, ranging from 30 to 46% and 4.3 to 8.4 months, respectively.

However, the length of time tumors responded to treatment (median duration of response) varied considerably, from 5.9 to 12.1 months^[13,18]^. Similarly, the median progression-free survival, which reflects how long patients live without their disease worsening, ranged from 2.9 to 8.8 months^[14,19]^.

Trastuzumab showed promise in shrinking tumors (antitumor activity) for some patients. One study observed a response rate of nearly 45% in a specific patient group, though responses in other groups were absent^[8]^. While the treatment did induce tumor shrinkage, the length of time tumors remained reduced (progression-free survival) was modest, averaging around 5–7 months^[8,9]^. Furthermore, the study looked at whether HER2 on the surface of cancer cells affected how much of the drug reached its target. This study found that HER2 levels did not influence how much of the drug was present in the bloodstream^[8]^. However, some studies suggested that patients with tumors that tested positive for HER2 benefited more from Trastuzumab combinations^[19]^. Nakamura *et al* (2021)^[13]^ also found that a blood test (ctDNA genotyping) could be as accurate as a tissue biopsy in identifying patients likely to respond well to Trastuzumab-based therapy.

Another study investigated whether Rat Sarcoma Virus (RAS) mutation impacted the effectiveness of Trastuzumab. This study found that Trastuzumab was effective even in patients with this mutation, even if they had already received other HER2-targeted therapies^[9]^. Mutations in specific genes, like RAS, influence how well a patient responds to treatment^[20]^.

While these combinations showed promise, they also caused side effects, with some being severe^[11]^. Common side effects included diarrhea, fatigue, nausea, and rash^[10,15]^. Less frequent but more serious side effects included gastrointestinal problems, bone marrow suppression (myelosuppression), nerve damage (neuropathy), and elevated liver enzymes^[18,20]^. Treatment-related deaths were reported in some studies, but not all^[14,21]^. Trastuzumab caused side effects, some of which were serious. Common side effects included lung problems, anemia, and decreased white blood cells^[8,9]^. More serious side effects included a lung condition called interstitial lung disease and even one treatment-related death^[9]^.

## Discussion

This study analyzed clinical trials investigating Trastuzumab used in combination with other drugs for cancer treatment. Trastuzumab combinations demonstrate efficacy, suggesting a viable strategy for HER2-positive CRC. However, several key points warrant further discussion regarding efficacy, safety, patient selection, and future research directions. Moreover, the high cost and limited accessibility of Trastuzumab-based combinations, particularly in low-resource settings, may hinder their widespread adoption.

The confirmed ORRs observed in these studies (22.2–46%) are encouraging, with some patients achieving complete remission. However, the variability in duration of response and progression-free survival highlights the need for further investigation into factors influencing treatment effectiveness. Future studies should explore the potential benefits of tailoring treatment regimens based on tumor biology and individual patient characteristics. The side effect profile of Trastuzumab combinations requires careful consideration. While common side effects like diarrhea and fatigue are manageable, the potential for severe adverse events necessitates close patient monitoring. Balancing treatment efficacy with patient tolerability remains crucial in optimizing treatment strategies.

Studies suggesting better outcomes in HER2-positive patients treated with Trastuzumab combinations support the targeted nature of this therapy. The potential of ctDNA genotyping as a reliable and less invasive method for identifying responders warrants further exploration to optimize patient selection for these therapies. Investigating the role of HER2 expression levels and other molecular markers in predicting response to different Trastuzumab combinations would be valuable for personalized treatment approaches.

The potential impact of RAS mutations on treatment response identified in some studies highlights the importance of exploring resistance mechanisms to Trastuzumab combinations. Understanding these mechanisms could lead to developing combination therapies that overcome resistance pathways and improve patient outcomes.

The observed variability in ORRs (22.2–46%) and progression-free survival (3.4–8.2 months) across studies may reflect patient heterogeneity, including differences in HER2 amplification levels, RAS mutation status, and prior treatment histories. Fu *et al* (2023) reported a higher ORR (33.3%) in RAS wild-type patients, suggesting molecular profiling could explain some differences. Study design also contributes, with phase II trials (Spiekman *et al*, 2024) showing higher ORRs due to controlled settings compared to retrospective studies (Yang *et al*, 2022). These factors show the need for standardized patient stratification.

The reported ORRs (22.2–46%) and median OS (up to 15.5 months with Trastuzumab deruxtecan, Yoshino *et al*, 2023) compare favorably to traditional chemotherapy regimens for HER2-positive CRC, which typically yield ORRs of 10–20% and OS of 12–14 months (FOLFOX). This suggests Trastuzumab combinations could offer a clinically meaningful improvement, potentially reducing reliance on toxic chemotherapies and enhancing quality of life for patients with limited advanced-stage options.

The encouraging results observed with Trastuzumab combinations in cancer treatment highlight the need for further exploration to improve their efficacy and broaden their clinical applications. There is a need to develop robust and reliable biomarkers beyond HER2 status to identify patients most likely to benefit from specific Trastuzumab combinations. Continued research on Trastuzumab combinations has the potential to translate into improved clinical outcomes for cancer patients. This could involve increased response rates, longer progression-free survival, and even cures for specific cancers.

Future research should prioritize large-scale, multicenter phase III trials to validate these findings. Specifically, prospective studies employing ctDNA genotyping with next-generation sequencing could identify responders, guided by protocols like those in Nakamura *et al* (2021). To address resistance, collaborative efforts should design preclinical models testing Trastuzumab with phosphoinositide-3-kinase/Ak strain transforming inhibitors, integrating bioinformatics to map resistance pathways.

Limitations include heterogeneity across studies (phase II vs. retrospective designs) and potential publication bias favoring positive outcomes. Additionally, some studies had relatively small sample sizes. Future research should involve larger, well-designed clinical trials with more extended follow-up periods to confirm these findings and assess Trastuzumab combinations’ long-term efficacy and safety. Investigating the optimal dosing schedules and exploring combinations with other targeted therapies or immunotherapies holds promise for further improving treatment outcomes.

## Conclusion

Trastuzumab combinations demonstrate efficacy for HER2-positive CRC, with future phase III trials essential to confirm efficacy and guide clinical practice. Clinical trials have demonstrated encouraging efficacy. Additionally, these combinations have shown the potential to improve OS in a subset of patients. However, several key questions remain to be addressed to optimize therapeutic strategies. A crucial area for future investigation is tailoring treatment regimens based on tumor biology and individual patient characteristics. This personalized approach can potentially improve response rates and the duration of response observed in clinical trials. Furthermore, a balance needs to be struck between treatment efficacy and patient safety. While common side effects associated with Trastuzumab combinations are often manageable, the potential for severe adverse events necessitates close patient monitoring.

Studies suggesting better outcomes in patients with HER2-positive tumors treated with Trastuzumab combinations support the targeted nature of this therapy. Further exploration of ctDNA genotyping as a reliable and less invasive method for identifying responders to Trastuzumab warrants investigation. This could optimize patient selection for these therapies and improve clinical outcomes. Additionally, investigating the role of HER2 expression levels and other molecular markers in predicting response to different Trastuzumab combinations would be valuable for developing personalized treatment approaches.

The potential impact of RAS mutations on treatment response, observed in some studies, highlights the importance of exploring resistance mechanisms to Trastuzumab combinations. A deeper understanding of these mechanisms could lead to developing combination therapies that overcome resistance pathways and improve patient outcomes.

## Data Availability

Data sharing is not applicable to this article as no datasets were generated.
